# Master of nursing programs in Australia: A desktop analysis

**DOI:** 10.1016/j.heliyon.2024.e35416

**Published:** 2024-07-30

**Authors:** Anita Dunn, Helena Harrison, Holly L. Northam, Ylona Chun Tie, Melanie Birks

**Affiliations:** a Nursing and Midwifery, College of Healthcare Sciences, Academy James Cook University, Douglas, 4811, Queensland, Australia; b Faculty of Health, School of Nursing, Midwifery and Public Health, University of Canberra, Bruce, 2617, Australian Capital Terrority, Australia

**Keywords:** Accreditation, Education, Graduate, Higher Education, Master of Nursing, Nursing

## Abstract

**Background:**

Master of Nursing (MN) programs serve as an important structured pathway to prepare the nursing workforce to develop advanced capabilities. Little research has been done to examine the key characteristics of MN programs in Australia and their responsiveness to meeting the health needs of the community.

**Aim:**

The aim of this desktop analysis is to provide a detailed overview of the key characteristics of MN programs in Australia.

**Method:**

A desktop analysis of MN programs in Australia utilising content analysis of publicly available information gathered from program websites of Australian Universities.

**Results:**

A total of 70 MN programs were analysed from the 28 that offered these programs. Nine categories were developed that describe the key characteristics of these programs: nomenclature and program focus, program outcomes, regulation and professional recognition, structure, work integrated learning, delivery mode, admission, and tuition fees. Inconsistencies were identified across all categories.

**Discussion:**

While Australian universities offer a diverse range of MN programs, inconsistencies across the programs can impact quality and intent and have implications for students, employees, and universities.

**Conclusion:**

There is a pressing need to ensure the quality of MN education is maintained and adequate to meet healthcare needs. The benefits and risks of professional regulation of all MN programs should be considered as a mechanism for enhancing the quality of nursing education.

## Highlights

• Master of Nursing programs prepare nurses with advanced capabilities.
• Master of Nursing programs prepare nurses to meet healthcare priorities.
• The structure and quality of these programs are often inconsistent.
• Inconsistencies risk educational goals, financial strain, employability anduniversity image.
• Robust regulation of programs may ensure graduates meet national healthcare needs.

## 1 Introduction

Registered nurses (RNs) occupy a crucial and evolving place in Australia's healthcare system. Nurses make up approximately 54 % or 349,589 of the total healthcare workforce, the largest body of healthcare professionals [1]. It is vital for public safety that the educational preparation of RNs undergoes rigorous examination [2]. The Schwartz report (2019), commissioned by the Australian Government to review nursing education in Australia, emphasised the need for contemporary, adaptative nursing, that focuses on clinical training, Indigenous health, technology integration, and interprofessional learning in nursing curricula. Extended educational pathways are an important strategy in meeting this need. In Australia, universities hold a strong international reputation for quality higher education, largely because of the regulatory processes determined by the Tertiary Education Quality Standards Agency (TEQSA) [3] and the Australian Qualifications Framework (AQF) [4]. These processes establish a minimum standard of higher education in Australia and support the maintenance of good education practice.

RNs graduating with a Bachelor degree are prepared for a generalist role at a novice to beginner level of practice and build their nursing expertise over time through practice across diverse settings [2]. While RNs with a Graduate Entry Master of Nursing (GEMN) are prepared at a master level, they commence nursing practice at a novice to beginner level. Conversely, RNs who undertake a Master of Nursing (MN) qualification commonly have more nursing experience and practice at a higher level. MN programs prepare RNs for more advanced generalist and/or specialist practice and roles and provide the opportunity for nurses to develop advanced capabilities in preparation to drive a progressive health agenda in nursing in Australia.

The exact number of Masters qualified RNs or their specific program of study are unknown, however two national surveys indicate that between 2016 and 2021 Masters qualified RNs increased from approximately 19 %–37 % [5,6]. Masters qualified RNs are described as leaders in their field [7] and expected to be equipped with advanced knowledge and specialised skills for leadership, research, education, and complex clinical practice within their designated area of work [4]. Despite this expectation, little research has been conducted into the programs that are designed to achieved these outcomes and ensure RNs are prepared for the advanced roles they will occupy.

RNs in advanced roles must be adequately prepared to meet Australia's emerging healthcare needs. Australia's healthcare system continues to face an increasing prevalence of complex and chronic diseases, an ageing population with co-morbidities [8], advancing technology and demanding healthcare environments. Pursuit of this goal is dependent on the availability of quality, relevant, and contemporaneous MN education. The accreditation of nursing programs serves an important role in ensuring the quality of nursing education supports the delivery of high-quality healthcare [9]. In Australia, the only MN programs that require accreditation are those that lead to initial registration as a RN (GEMN program) or endorsement as a Nurse Practitioner (NP) [9]. While these accreditation standards are mandated by the Health Practitioner Regulation National Law Act, 2009 [10], accreditation processes established by professional colleges, such as the Australian College of Mental Health Nurses (ACMHN) while not mandated, provide a consistent approach to the structure and content of specialist programs that contribute to the quality of this education [11].

Education programs for RNs seeking a MN qualification need to be accessible. Many nurses who complete ongoing education often do so while maintaining employment and family commitments [12]. To increase the availability of education for nurses, flexible delivery of programs has been a mainstay of nursing education for decades. The recent explosion of online learning has increased opportunities for students wishing to engage in further study [13]. Geographic location is no longer a barrier to educational opportunities. Nurses can secure a MN qualification from anywhere in the international environment, providing they have the time, technological literacy, and resources to meet the associated costs of study [14].

What can prove a barrier to prospective students is cost. In a market-driven model, universities target full fee paying domestic and international students with the latter having become a significant source of revenue for universities over the past two decades [15]. Completing a MN program requires commitment and significant personal and financial investment. Nurses rely on accessible, clear information provided to them by individual universities when seeking to make decisions about their further education.

The aim of this desktop analysis is to provide a detailed overview of the key characteristics of MN programs in Australia, the accessibility of this information to prospective students, and the implications of the structure, content, and delivery of these programs for the graduates and the healthcare system in Australia.

## 2 Method

**Fig. 1:**
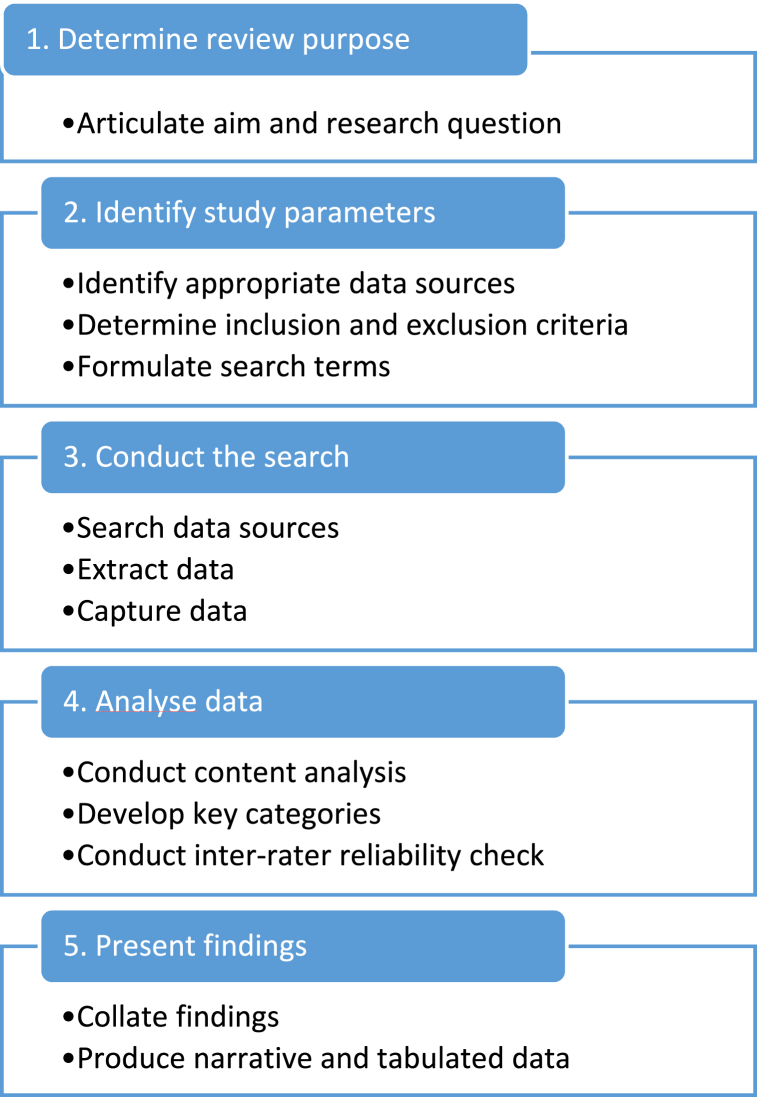
Desktop analysis process adapted from Turoń and Kubik [16].

A five step desktop analysis was selected for this study. A desktop analysis, often referred to as desk research, is a process that uses existing sources to obtain information about a particular topic without field work. Desktop analysis is a time efficient, economical process for analysing secondary data and documents on a large scale [16]. This method enables the collection of a breadth of data from a broad selection of sources and it does not usually require approval from a research ethics board. This study relied on sourcing existing information that was publicly available from Australian University websites. The process used in this desktop analysis was inspired by the method outlined by Turoń and Kubik [16] to ensure a flexible, yet systematic exploratory approach to examining MN programs in Australia. The steps used in the desktop analysis are presented at Fig. 1.

In the first step the review purpose was determined. The research aim was drawn from a gap analysis of existing literature about MN programs, informed by the researchers' knowledge and expertise in teaching and researching in higher education. In the second step, the study parameters were identified. A list of all universities in Australia [17] was collated as the main source of information about MN programs. An inclusion and exclusion criteria was developed (Table 1) and key search terms identified. These included “Master of Nursing” and “Postgraduate Nursing”. In step three the search was conducted in March 2023 by the first named author by entering the key search terms on each university's website. Information about MN programs that met the inclusion criteria was entered into a master spreadsheet prepared for data management. In the fourth step, content analysis was used to analyse the information and identify key characteristics of MN programs. Content analysis is a flexible, inductive, data driven approach to data analysis [18] that allows for a systematic yet non-linear approach to understanding the patterns in the data. As the study progressed, categories were developed with other members of the research team providing inter-rater reliability confirmation of the findings. Categories were finalised when consensus was reached that the key characteristics of the MN of programs in Australia had been captured. In step five, the findings were collated and presented in narrative and tabulated form. The narrative data and summary tables are presented below. The master spreadsheet containing the compiled comprehensive data set is available in the Supplementary File.

**Table 1:** Inclusion and exclusion criteria.

Inclusion Criteria	Exclusion Criteria
Designed for registered nurses, or leading to registration as a nurse	Undergraduate programs
	Non-nursing programs
Graduate program at Masters level	Post-graduate certificate or diploma
Offered by Australian universities	Offered by international universities

## 3 Findings

Websites of the forty-one Australian universities were individually searched. Twenty-eight were found to offer a total of 70 MN programs. Programs were offered in all states and territories except for the Australian Capital Territory (ACT). Nine categories were developed to describe the main characteristics of MN programs. These are presented in Table 2.

**Table 2:** Categories.

Category
Nomenclature and program focus
Program outcomes
Regulation and professional recognition
Structure
Work Integrated Learning
Delivery mode
Admission
Tuition fees

## 4 Nomenclature and program focus

A diverse range of nomenclature was used to identify MN programs. These are presented in Table 3. Nomenclature included a title and in many cases a subtitle which indicated the program focus. There were a total of 13 GEMN programs, however, nomenclature that indicated these were graduate entry programs was varied. Main titles of GEMN programs included Master of *Nursing* (n = 3) and Master of *Nursing Practice* (n = 4), *Science* (n = 1) *or Studies* (n = 1), Master of *Clinical Nursing* (n = 2) and double titles to indicate the program was a double degree program (n = 2). Subtitles were equally variable and included pre-registration (n = 4), graduate entry (n = 2), and entry to practice (n = 1). Six program titles provided no indication they were graduate entry programs. There were 11 NP programs that enabled graduates to seek endorsement as a NP with the Australian Health Practitioner Agency (Ahpra). Titles of these programs also varied. These ranged between Master of *Nursing* with a subtitle Nurse Practitioner (n = 4) and NP (n = 1), Master of *Nurse Practitioner* with no subtitle (n = 5) and one with a subtitle of Mental Health (n = 1).

**Table 3:** Nomenclature and program focus.

Name	Focus of program	Number
Master of Nursing	GEMN	1
Master of Nursing (Graduate Entry)	GEMN	1
Master of Nursing – Entry to Practice	GEMN	1
Master of Nursing Practice (Pre-registration)	GEMN	2
Master of Nursing Practice	GEMN	2
Master of Nursing Science	GEMN	1
Master of Nursing Studies	GEMN	1
Master of Clinical Nursing	GEMN	1
Master of Clinical Nursing (Graduate Entry)	GEMN	1
Bachelor of Arts/Master of Nursing (Pre-registration)	GEMN	1
Bachelor of Science/Master of Nursing (Pre-registration)	GEMN	1
		**Total GEMN: 13**
Master of Nursing (Nurse Practitioner)	NP	4
Master of Nursing (NP)	NP	1
Master of Nurse Practitioner	NP	5
Master of Nurse Practitioner (Mental Health)	NP	1
		**Total NP: 11**
Master of Nursing	OTHER	10
Master of Advanced Nursing	OTHER	2
Master of Advanced Nursing Practice	OTHER	3
Master of Clinical Nursing	OTHER	3
Master of Nursing Science	OTHER	1
Master of Nursing (online)	OTHER	1
Master of Nursing (Acute Care)	OTHER	1
Master of Nursing (by research)	OTHER	1
Master of Nursing (Clinical Leadership)	OTHER	1
Master of Nursing (Coursework and Research)	OTHER	1
Master of Nursing (Critical Care)	OTHER	1
Master of Nursing (Diabetes Management and Education)	OTHER	1
Master of Nursing (Mental Health)	OTHER	1
Master of Nursing (Professional Studies)	OTHER	1
Master of Nursing (Research)	OTHER	1
Master of Nursing (with Specialisations)	OTHER	1
Master of Nursing International	OTHER	1
Master of Nurse Education	OTHER	1
Master of Cancer and Haematology Nursing	OTHER	1
Master of Clinical Nursing (Specialisation)	OTHER	1
Master of Emergency Nursing	OTHER	1
Master of Intensive Care Nursing	OTHER	1
Master of Mental Health Nursing	OTHER	6
Master of Health (Advanced Nursing)	OTHER	1
Master of Health (Child and Family Health Nursing)	OTHER	1
Master of Health (Mental Health Nursing)	OTHER	1
Master of Philosophy (Nursing)	OTHER	1
		**Total Other: 46**

In the remaining 46 programs, similar variation was noted in the titles and subtitles. No subtitles indicated a generic focus on nursing. Some titles suggested a type or level of practice, such as Master of *Nursing* (n = 10), *Advanced Nursing* (n = 2) or *Advanced Nursing Practice* (n = 3), *Clinical Nursing* (n = 3), and *Nursing Science* (n = 1). One NP program included a subtitle to indicate online delivery mode. Nomenclature of the remaining programs (n = 26) indicated a focus on various areas of nursing including mental health, acute care, intensive care, nurse education, critical care, diabetes management and education, child and family nursing, emergency nursing, cancer and haematology nursing, clinical leadership, and international nursing. Three of these programs were titled Master of *health* and one titled Master of *philosophy*, all with a nursing focused subtitle.

## 5 Program outcomes

Program learning outcomes were found for 33 of the 70 programs. These outcomes largely focused on the expansion of advanced skills, knowledge, communication, leadership, research, cultural diversity, and critical thinking skills for practice. Learning outcomes appeared to be informed by the AQF educational framework [4] and for accredited programs, regulatory standards. In some programs, the synopsis focused on outcomes related to increased employment opportunities, particularly in research, education, and/or leadership and management. Other less citied employment opportunities related to administration and consultancy, aged care, rural and remote nursing, policy development, chronic disease management, trauma, and disaster management.

## 6 Regulation and professional recognition

The regulation and professional recognition status of the programs are presented in Table 4. All 28 universities offering MN programs held self-accreditation status against the TEQSA Standards. This information was not readily evident on most university websites with only three universities stating their TEQSA accreditation status in their MN program handbook designed to provide information about the program of study.

**Table 4:**
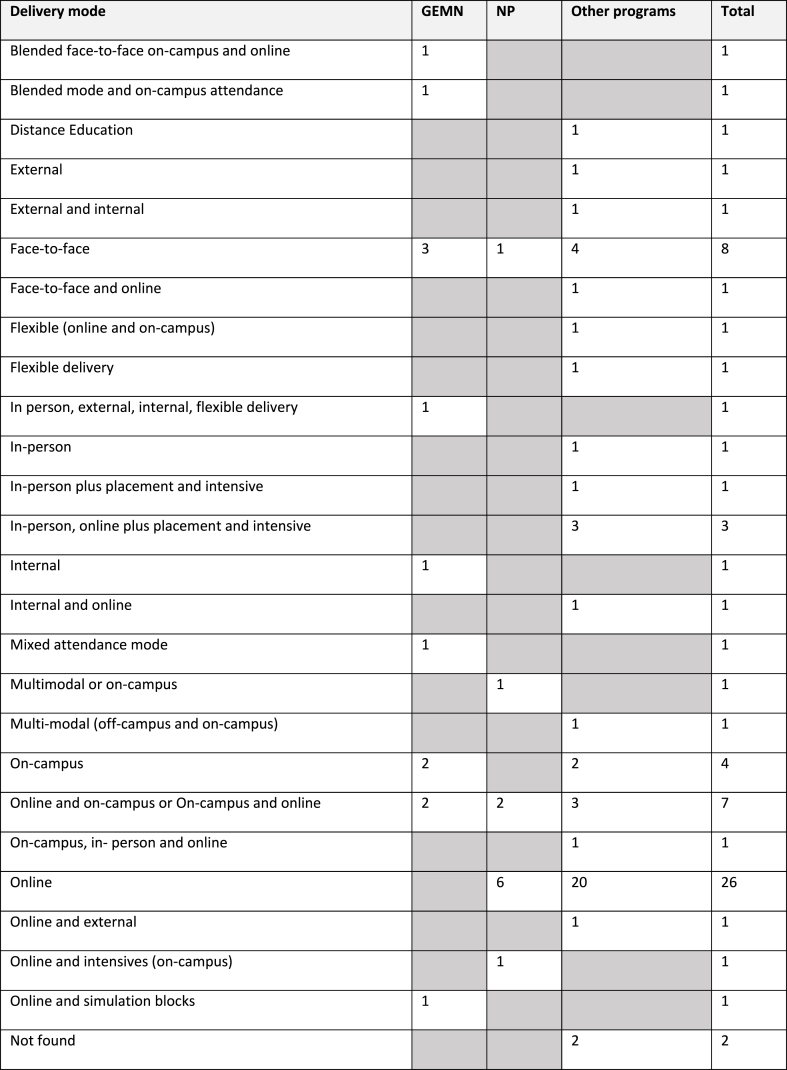
Delivery mode.

All 13 GEMN and 11 NP programs indicated that the program met the Australian Nursing and Midwifery Accreditation Council (ANMAC) requirements for accreditation [9] on their program websites. Four of the Mental Health programs indicated they were developed against the mental health standards established by the ACMHN [11]. This recognition signified that the program met criteria that aligned with the national framework for postgraduate (PG) mental health nursing education and that graduates could expect to use the completed qualification as part of the credentialing process to become a Mental Health Nurse. No other form of professional recognition was found for the remaining 42 MN programs included in this desktop analysis.

## 7 Structure

The structure of the programs varied and were subcategorised into subjects, credit allocation and duration.

### 7.1 Subjects

Subjects were detailed for 68 of the 70 MN programs, with these including compulsory and/or elective subjects. The 13 GEMN and 10 NP programs contained exclusively compulsory subjects, one NP program incorporated two elective subjects. Compulsory subjects in these programs were designed to ensure that programs met accreditation requirements, and that graduates achieved the associated standards for practice***.*** NP programs contained five to 12 compulsory subjects. GEMN programs contained between nine and 18 compulsory subjects. The remaining 42 programs contained a variety of compulsory and elective subjects. Compulsory subjects ranged from one to 12 across those programs, with between one and four elective subjects.

### 7.2 Credit allocation

Credit point allocation for subjects varied across the programs, according to the university credit point system, thereby making prima facie comparison difficult. One program required eight credit points for program completion, while others up to 400 credit points. In programs that allocated a higher number of total credit points, there tended to be more subjects to complete. As each university had self-accrediting status with TEQSA, it can be expected that all programs met the minimum workload expectations for an AQF level nine qualification.

### 7.3 Duration

Most programs could be completed within one to two years full time (FT), part time (PT) or part time equivalent (PTE). Double degree programs could be completed over four years FT, or PTE. Eight of the NP, one Master of Clinical Nursing, and one Master of Nursing program, were only available on a part time basis. One GEMN and the Master of Nursing International were only available full time. It should be noted that students on international study visas were restricted to enrolling in eligible programs on a full-time basis to fulfil their visa requirements [19].

## 8 Work integrated learning

In addition to the virtual and/or physical attendance requirements, some programs included a work integrated learning (WIL) component. Consistent with ANMAC accreditation requirements, GEMN programs prescribed a minimum of 800 clinical placement hours, with NP programs, a minimum of 300 supernumerary hours [9]. For all other programs, there was significant variation in WIL requirements. Some programs did not require any WIL (n = 33), others required relevant industry employment between 0.5 and 2 days, or 16–18 hours a week (n = 9) and some required observational (n = 1) or clinical placement (n = 3).

## 9 Delivery mode

Delivery modes were identified by the terms and descriptions stated by each university. Twenty-five different terms were used to describe delivery mode. The terms are presented in Table 4. While terms and descriptions varied across and within MN programs, limited information was provided to clarify what the terms represented. Delivery mode was often buried in course information and/or subject outlines. The majority of programs were described as being delivered online (n = 26), face-to-face (n = 8), on-campus and online (n = 7), with variations noted within subjects. The remaining programs used one or a combination of terms to describe delivery modes, which at times, varied between subjects. These included blended or blended mode, distance education, external, internal, flexible, flexible delivery, in-person, online plus placement and intensive, mixed mode, multi-modal, and on-campus. In some instances, programs were presented as being delivered online, however further exploration revealed face-to-face or on campus and clinical placement requirements. Program delivery mode was not discernible for two programs.

## 10 Admission

All programs included admission requirements accessible via a webpage and/or a program handbook. Admission requirements were influenced by a program's accreditation status and whether the applicant was a domestic or international student. Forty programs were open to domestic and international students, with three of these limiting international student enrolment to specific majors. Twenty-eight programs were only open to domestic students, which included all the NP programs. One program was open to international students only and one program did not specify this information.

With the exception of GEMN, admission to MN programs required students to have Division One nursing registration with Ahpra or an approved nursing board. Applicants for all programs were required to demonstrate they met the accrediting body and/or university's English language requirements. GEMN programs required students to hold a bachelor degree completed within the last 10 years, and a minimum grade point average (GPA) between 4.0 and 5.0. Some GEMN programs stipulated that students with a non-science degree must complete compulsory anatomy and physiology subjects prior to admission to the program. NP program applicants were required to hold minimum educational and experiential qualifications consistent with accreditation requirements [9].

## 11 Tuition fees

Tuition fees were categorised into domestic commonwealth supported places (CSP) (government subsidized tuition fees), domestic full fee paying (FFP) and international FFP. CSPs were identified for 46 of the 70 MN programs and ranged from $4120 to $16,950 per annum over the duration of the program. FFP options were found for 38 programs with fees ranging from $11,650 to $48,500 per annum. International student fees were indicated in 28 of the 70 programs and ranged between $16,800 to $52,000 per annum. In some instances, fees were either not found or not available. Tuition fees did not appear to vary in accordance with delivery mode.

## 12 Discussion

The purpose of this desktop analysis was to provide a detailed overview of the key characteristics of MN programs in Australia. Findings illustrate the wide variety of MN programs offered by Australian universities, with a commensurate degree of variation in how these programs are structured and delivered. Consistency is integral to quality in higher education [20]. Consistency and quality over time yields a dual reciprocal benefit; students have more trust and confidence that their university can meet their educational and career aspirations, while universities benefit in building their reputation and brand. As a result, excellence in program outcomes attract more students, funding, and distinguished academics, thereby reinforcing Australia's global reputation in education.

Given the regulatory standards determined by TEQSA, a high degree of consistency in how MN programs are designed and communicated should be expected. Higher education institutions can apply for the authority to self-accredit programs, fields and/or levels of education [3]. With self-accreditation status, Australian institutions have the autonomy to determine the design, delivery, and content of their education programs and how this information is communicated to potential students. With this comes the responsibility to establish robust quality assurance processes and risk management strategies to ensure adherence with the regulatory requirements. Such processes also help to ensure a level of consistency in the education provided by higher education institutions.

All universities included in this study had self-accreditation status awarded by TEQSA, however, despite the quality mandates associated with this process, the most striking finding of this review was the lack of consistency evident in how MN programs were constructed and communicated. Accessing and clarifying information about the programs, their structure and delivery differed across each university. Prospective students most often rely on university websites for program information [21]. Information provided by this medium is arguably a reflection of the program itself. Where consistency is lacking in program information, students may find it difficult to identify an appropriate, quality program of study, transfer credits between programs with differing structures and expectations, or could unwittingly enrol in programs not suited to their personal situation and financial capacity. Furthermore, employers may be uncertain of graduate capabilities, and universities perceived to be offering less standardised educational quality.

Inconsistency was particularly evident in the nomenclature and program outcomes of the MN programs. According to the AQF, all programs at a Master level must include the term ‘Master of … *’* in the program title ([4], p. 61). While all programs included in this review met this requirement, nomenclature to indicate program focus was inconsistent. Furthermore, overall program learning outcomes were not found for over half the programs included in this study. Program learning outcomes are a fundamental component of the TEQSA Higher Education Standards (HES) Framework (Threshold Standards) 2021 [22]. Learning outcomes are essential for mapping curriculum, assessments, and student achievement in nursing [23], and for understanding outcomes from engaging in study. While this desktop analysis does not suggest non-compliance with the threshold standards, when a program focus and/or program learning outcomes are not included, students are hampered in making an informed decision about the program they wish to enrol. Enrolling in a program that is unsuitable can have time and financial implications. Visibility of program focus and learning outcomes can help students avoid such consequences.

Terminology to describe program delivery was another area of inconsistency in the material examined. Program handbooks or websites provided limited information to clarify what the different terms meant. While online and blended delivery modes have several benefits, defining related terminology has a history of being problematic [24,25]. In MN programs, online delivery mode has increased access to this education and become a strong marketing tool for universities to attract a diverse cohort of students, offering flexibility study schedules without being limited by geographical location [12]. Without a consistent approach to defining and communicating delivery mode, students and employers may not have a clear understanding of the expectations of where or how learning is to be completed, and in which mode. Concomitantly, findings identified a large variation in the number of subjects and credit allocations in MN programs. A program with less subjects and a lower credit allocation, may be perceived as having a lower workload and be less time consuming to complete. If there is a mismatch between the university and student's perception of delivery mode and subject completion requirements, students may unintentionally enrol in a program that does not suit their learning needs and/or personal and financial situation, and therefore unable to fully commit to the required workload or attendance mode. This can place students at risk of attrition, underperformance, and personal and financial strain. To avoid such consequences, it is crucial that universities provide clear and comprehensible information about MN programs, particularly in respect of costs, delivery modes, subject completion requirements, workload, and attendance expectations. Standardising how information about these program elements is described and communicated may provide clarity and reduce confusion for prospective students.

Evidence suggests WIL is important for enhancing learning, employability, and student capital [26,27]. WIL was evident in several MN programs, however only consistently applied in programs with ANMAC accreditation or professional recognition from the ACMHN. In other MN programs, WIL varied from no requirement to industry employment, observational and supernumerary experience, or clinical placement. With such variation in graduate capabilities, students, employers, and healthcare consumers cannot be assured of the quality and consistency in graduate capabilities on completion of MN programs.

Tuition fees were also found to vary considerably across the MN programs reviewed in this study. CSPs offered for MN programs are limited and many reserved for NP and GEMN programs. The majority of other programs required full fee paying or international fees, which are typically at least two to three times the cost of government funded CSPs. This finding supports Schwartz [2] review into nursing education in Australia, that found many PG nursing programs require students to take on high levels of debt. Ng et al. [28] and Macdiarmiad et al. [29] indicate that a key factor for students deciding to enroll in PG education is tuition fees. Given the potential costs of study, students must weigh up the significant debt associated with MN programs in higher education against the desire to build the advanced nursing capabilities that these programs offer.

Despite the findings that highlighted inconsistencies in MN programs in Australia, some consistency was found, particularly in program duration, enrolment status, and career opportunities. These factors reflect the influence of the TEQSA HES (Threshold Standards) 2021 [22] and the AQF [4] requirements. With a clear indication of how programs can enhance career aspirations, students can be more confident that their studies will help them to achieve their PG objectives [14]. The highest level of consistency was found in GEMN and NP programs and those with professional recognition. These programs are developed based on established higher education, regulatory and nursing standards [9,11]. In these cases, greater consistency was found in nomenclature, core subjects and delivery, WIL, and admission requirements, suggesting that where the design and delivery of programs requires adherence to regulatory processes, greater consistency ensues. Highly regulated MN programs prepare RNs with advanced capabilities and ensure greater certainty in the expectations and output of graduates [30,31]. It can be concluded, therefore, that regulation across all MN programs could help to ensure graduates are adequately and equally equipped to meet national healthcare needs and priorities.

## 13 Implications for practice

The health system is now under greater pressure than ever to provide safe and quality healthcare that meets the changing needs of healthcare consumers. MN programs need to be flexible and adaptable to support nurses to meet these needs. The findings identified in this study suggests that graduates of MN programs may be at risk of being inadequately prepared to meet new and evolving healthcare demands, particularly in key national health priority areas such as chronic disease, diabetes management, primary healthcare, rural, remote and First Nations health [32]. The lack of MN programs in these areas reflects findings from the Schwartz [2] review that nursing roles are only in their infancy in terms of their contribution to health promotion and the prevention of chronic illness.

Given the expected outcomes of a masters level of education [3,4] and the imperative to ensure graduates of MN programs are prepared as leaders who can address the evolving complexity of healthcare in Australia, addressing inconsistencies such as those identified in MN programs in this study is essential. Accrediting authorities significantly influence the quality and educational outcomes of higher education programs. Increasing professional regulation across all MN programs could help overcome the current limitations in MN nursing education. Ensuring greater consistency in MN Programs inclusions, outcomes, and how these are communicated to the public, is reliant on nursing bodies and specialty areas of practice collaborating to provide standards for benchmarking across institutions, similar to those used for accreditation or professional recognition purposes. Further research about the impact of MN programs would assist in determining effective program delivery and necessary content, while providing evidence to establish benchmarking and regulatory standards.

## 14 Limitations

In any desktop analysis, only pre-existing, accessible data is gathered. The ability to confirm the accuracy, quality and specificity of pre-existing data can be limited, which can impact the credibility of the findings. This study relied on sourcing current information from Australian university websites. There is broad diversity in how, where, and what information is provided about MN programs despite these programs being subject to regulatory standards. Variable terms and locations meant that data was at times convoluted and difficult to access. Where such data may have been available but not easily obtainable, it could not be included in the study. Establishment of clear inclusion and exclusion criteria and inter-rater reliability checks were key to reducing identified limitations.

## 15 Conclusion

In Australia's dynamic healthcare landscape, there is a pressing need to ensure quality of nursing education is maintained and adequate to meet healthcare needs. RNs are the forefront of healthcare delivery and will require advanced capabilities to fulfill current and future healthcare responsibilities. This desktop analysis has highlighted the need for greater consistency across MN programs in Australia and better alignment of these programs to national healthcare priorities. Strengthening consistency and clarity in the delivery, structure, and content across programs will go some way to ensuring students graduate with the required capabilities to fulfill their advancing role.

## Data availability

Data is included in the article and supplementary material and referenced in article.

## CRediT authorship contribution statement

**Anita Dunn:** Writing – review & editing, Writing – original draft, Visualization, Validation, Software, Resources, Project administration, Methodology, Investigation, Formal analysis, Data curation, Conceptualization. **Helena Harrison:** Writing – review & editing, Writing – original draft, Visualization, Validation, Supervision, Resources, Project administration, Methodology, Investigation, Formal analysis, Data curation, Conceptualization. **Holly L. Northam:** Writing – review & editing, Validation, Methodology, Conceptualization. **Ylona Chun Tie:** Writing – review & editing, Supervision, Conceptualization. **Melanie Birks:** Writing – review & editing, Visualization, Validation, Supervision, Methodology, Investigation, Conceptualization.

## Declaration of competing interest

The authors declare that they have no known competing financial interests or personal relationships that could have appeared to influence the work reported in this paper.
